# Emerging roles of long non‐coding RNAs in neuropathic pain

**DOI:** 10.1111/cpr.12528

**Published:** 2018-10-25

**Authors:** Zheng Li, Xingye Li, Xin Chen, Shugang Li, Idy H. T. Ho, Xiaodong Liu, Matthew T. V. Chan, William K. K. Wu

**Affiliations:** ^1^ Department of Orthopaedic Surgery, Peking Union Medical College Hospital Chinese Academy of Medical Sciences and Peking Union Medical College Beijing China; ^2^ Department of Orthopedic Surgery, Beijing Jishuitan Hospital, Fourth Clinical College of Peking University Jishuitan Orthopaedic College of Tsinghua University Beijing China; ^3^ Department of Anaesthesia and Intensive Care The Chinese University of Hong Kong Hong Kong Hong Kong Special Administrative Region Hong Kong; ^4^ State Key Laboratory of Digestive Diseases, LKS Institute of Health Sciences The Chinese University of Hong Kong Hong Kong Hong Kong Special Administrative Region Hong Kong

**Keywords:** diabetic peripheral neuropathy, lncRNAs, nerve pain, neuroinflammation, spinal cord pain

## Abstract

Neuropathic pain, a type of chronic and potentially disabling pain resulting from primary injury/dysfunction of the somatosensory nervous system and spinal cord injury, is one of the most intense types of chronic pain, which incurs a significant economic and public health burden. However, our understanding of its cellular and molecular pathogenesis is still far from complete. Long non‐coding RNAs (lncRNAs) are important regulators of gene expression and have recently been characterized as key modulators of neuronal functions. Emerging evidence suggested that lncRNAs are deregulated and play pivotal roles in the development of neuropathic pain. This review summarizes the current knowledge about the roles of deregulated lncRNAs (eg, KCNA2‐AS, uc.48+, NONRATT021972, MRAK009713, XIST, CCAT1) in the development of neuropathic pain. These studies suggested that specific regulation of lncRNAs or their downstream targets might provide novel therapeutic avenues for this refractory disease.

## 1 INTRODUCTION

Neuropathic pain, a type of chronic and potentially disabling pain resulting from primary injury/dysfunction of the somatosensory nervous system, is a serious public health issue with an estimated prevalence of 7%‐10%.^1^, ^2^, ^3^ Specific causes include postherpetic neuralgia, trigeminal neuralgia, painful diabetic peripheral neuropathy, cancer‐related neuropathic pain and traumatic neural injury/compression.^4^, ^5^ Genetic component, such as single nucleotide polymorphisms in *IL10*, also plays a contributory role.^6^ Both peripheral sensitization and central sensitization take part in the development of neuropathic pain, wherein deregulated neuronal firing and glial function alter nociceptive signalling and processing, leading to a lowered pain threshold.^7^ Clinically, neuropathic pain is difficult to treat, with all existing therapies (eg, anticonvulsants acting at calcium channels, tricyclic antidepressants, serotonin‐noradrenaline reuptake inhibitors, topical lidocaine, opioids) variably alleviating the pain without fully addressing the underlying pathophysiology.^8^ Therefore, it is crucial to identify novel molecular targets for developing mechanism‐driven treatments that can effectively kerb this disease.

Long non‐coding RNAs (lncRNAs) are a class of regulatory RNAs that are longer than 200 nucleotides in length yet without protein‐coding potential.^9^, ^10^, ^11^, ^12^ Through regulating gene expression at multiple levels (eg, DNA methylation, histone modification, recruitment of transcriptional factors, sponging microRNAs, regulation of mRNA stability and splicing), lncRNAs play pivotal roles in different cellular processes, including cell proliferation,^13^, ^14^, ^15^, ^16^ differentiation,^17^ apoptosis, autophagy,^18^ cellular senescence,^19^ migration and invasion.^20^ Strikingly, 40% of lncRNAs are expressed specifically in the brain.^21^ There are growing number of studies reporting that lncRNA expression is responsive to neuronal activity and injury, in which lncRNAs modulate nervous system development and synaptic plasticity by regulating neuronal outgrowth, differentiation as well as synapse formation and function.^21^, ^22^, ^23^ Importantly, lncRNA deregulation has been reported in different neurological and psychiatric conditions.^24^, ^25^ Recently, emerging evidence suggested that lncRNAs could play crucial roles in development of neuropathic pain.^26^, ^27^, ^28^ In this article, we review current knowledge about the deregulation of lncRNAs in neuropathic pain in relation to their cellular and molecular functions. The potential utilities of these lncRNAs or their downstream mediators as therapeutic targets for neuropathic pain are also discussed.

## 2 LNCRNA EXPRESSION PROFILING IN NEUROPATHIC PAIN

LncRNA expression profiling by whole transcriptome shotgun sequencing, PCR array or microarray followed by validation of candidate lncRNAs by reverse transcription‐quantitative PCR (RT‐qPCR) is the most common approach to identify and validate differentially expressed lncRNAs in specific disease states.^29^ Owing to ethical concerns in obtaining human neural tissues for lncRNA profiling, the samples analysed in neuropathic pain studies are usually restricted to animal tissues.

Spinal nerve ligation (SNL) is a common experimental approach to induce neuropathic pain, in which the animals experience persistent pain characterized by mechanical allodynia and heat hyperalgesia. Jiang and colleagues^30^ performed mRNA and lncRNA microarrays in mice undergone SNL to identify deregulated lncRNAs in the spinal cord. Using the criteria of fold change >2 and *q*‐value <0.05, they identified a total of 511 differentially expressed (145 downregulated and 366 upregulated) lncRNAs as compared with the sham‐operated group. The deregulation of two selected upregulated (Speer7‐ps1 and uc007pbc.1) and two downregulated (ENSMUST00000171761 and ENSMUST00000097503) lncRNAs were further confirmed by RT‐qPCR. Bioinformatic analysis revealed 39 differentially expressed lncRNA‐mRNA pairs, among which 32 pairs exhibited concordant direction of deregulation. These lncRNA‐mRNA pairs were involved in pathways, such as Toll‐like receptor signalling, calcium signalling and peroxisome proliferator‐activated receptor signalling.^30^ This study shed new light on lncRNA deregulation and their potential downstream pathways in the development of SNL‐induced neuropathic pain.

Spared nerve injury (SNI), which involves ligation of two of the three branches of the sciatic nerve, produces symptoms of peripheral neuropathic pain. Zhou and colleagues^31^ performed the whole transcriptome shotgun sequencing to profile the expression of non‐coding RNAs, including lncRNAs, microRNAs and circular RNAs, in relation to that of mRNAs in the spinal cord of Sprague‐Dawley rats which sustained SNI. A total of 134 lncRNAs, 12 microRNAs, 188 circular RNAs and 1066 mRNAs were found to be significantly deregulated on day 14 post‐SNI. The deregulation of two selected lncRNAs (XLOC_021333 and Rn50_8_0646.1) was validated by RT‐qPCR. The lncRNA‐microRNA‐mRNA network was then re‐constructed, illustrating the potential involvements of ribosome, phosphatidylinositol 3‐kinase‐Akt signalling pathway, focal adhesion and extracellular matrix‐receptor interactions in the pathogenesis of SNI‐induced neuropathic pain.^31^ The same group of investigators later published another study using the same Sprague‐Dawley rat SNI model to delineate the deregulation of lncRNAs and signalling pathways in the spinal cord in relation to the temporal course of neuropathic pain development.^32^ Using the mouse SNI model, Liu and colleagues^33^ identified 1200 and 739 differentially expressed lncRNAs and mRNAs, respectively, in the ipsilateral spinal cord. Interestingly, minocycline (a second‐generation tetracycline antibiotic used for the treatment of neuropathic pain) significantly attenuated the deregulation of lncRNAs and mRNAs and SNI‐induced neuropathic pain in mice. The upregulation of three lncRNAs, namely ENSMUST00000146263, ENSMUST00000174263 and NR_015491, and their downregulation by minocycline were further confirmed by RT‐qPCR.^33^

By meta‐analysing microarray data, Raju and colleagues identified differentially expressed non‐coding RNAs, including eight long‐intergenic non‐coding RNAs, 12 antisense RNA and 56 pseudogenes, in dorsal root ganglions (DRGs) of rats following sciatic nerve injury. Further contextual analysis of the differentially expressed pseudogenes revealed their associations with neurodegeneration and/or neurogenesis.^34^

The above‐mentioned animal and bioinformatic studies strongly suggested that lncRNA deregulation is closely linked to neuropathic pain, in which the differentially expressed lncRNAs might take part in the pathogenesis (Table 1).

**Table 1:** Long non‐coding RNA expression profiles in neuropathic pain

Num	Method	Sample	Microarray filtering criteria	Upregulated	Downregulated	References
1	Microarray RT‐PCR	Mice spinal nerve ligation	of fold change > 2 and *q*‐value < 0.05	366 lncRNAs	145 lncRNAs	30
2	Whole transcriptome shotgun sequencing	Rats spared nerve injury	*P* < 0.05; fold change > 2	134 lncRNAs XLOC_021333 Rn50_8_0646.1	No mention	31
3	Whole transcriptome shotgun sequencing	Rats spared nerve injury	*P* < 0.05; fold change > 2	1200 lncRNAs ENSMUST00000146263, ENSMUST00000174263, NR_015491	No mention	32

lncRNAs, long non‐coding RNAs.

## 3 FUNCTIONAL ROLES OF LNCRNAS IN NEUROPATHIC PAIN

Emerging studies have highlighted the molecular functions of lncRNAs in the pathogenesis of different neurological diseases. One of the most important functions of lncRNAs is to modulate gene expression. LncRNAs may act as scaffolds of transcriptional and epigenetic protein complex, which can interact with specific genomic loci to modulate gene transcription. LncRNAs may also affect RNA processing by directly interacting with target mRNAs. Alternatively, lncRNAs may “sponge” microRNAs, leading to the disinhibition of their downstream target genes. The functional characterization of differentially expressed lncRNAs in neuropathic pain is just emerging. Herein, specific lncRNAs with their functions and molecular mechanisms characterized in neuropathic pain are discussed in detail.

### 3.1 KCNA2‐AS

Nerve injury is known to reduce the expression of the voltage‐dependent potassium (Kv) channel subunit Kcna2 through promoter hypermethylation, thereby decreasing total Kv current and enhancing neuronal excitability.^35^ Zhao and colleagues^26^ identified a novel lncRNA known as KCNA2‐AS whose sequence is complementary to Kcna2 RNA. The expression of KCNA2‐AS could be detected in DRG neurons of rat, mouse, monkey and human. In rats, most KCNA2‐AS–positive neurons expressed low levels of Kcna2 protein, suggesting a possible negative regulation of Kcna2 by this lncRNA. The authors further showed that SNL enhanced the expression of KCNA2‐AS through the transcriptional activator myeloid zinc finger protein 1 (MZF1), with concomitant downregulation of Kcna2 in the ipsilateral DRG. Functionally, microinjection of KCNA2‐AS into DRG reduced total Kv current and increased the excitability of DRG neurons, producing neuropathic pain symptoms. Concordantly, blockade of KCNA2‐AS function by adenovirus‐mediated delivery of Kcna2 sense fragment produced opposite effects.^26^ These data suggested that KCNA2‐AS and its upstream regulator MZF1 represent novel therapeutic targets in neuropathic pain.

### 3.2 uc.48+

Diabetic peripheral neuropathy is a major cause of neuropathic pain. Activation of P2X purinoceptors expressed on first‐order sensory neurons by ATP released from damaged or inflamed tissues has been shown to mediate pain hypersensitivity in diabetic peripheral neuropathy.^36^ A study by Wang and colleagues^27^ demonstrated that the expression levels of a lncRNA known as uc.48+ were elevated in the diabetic rat DRG, whose knockdown with small interference RNA (siRNA) reduced diabetes‐induced mechanical allodynia and thermal hyperalgesia. Mechanistically, the upregulation of P2X_3_ and its downstream ERK pathway in DRG of diabetic rats could be abrogated by uc.48 + siRNA, suggesting that uc.48+ is an upstream positive regulator of P2X_3_. Moreover, knockdown of uc.48+ alleviated the pro‐inflammatory signals in DRG of diabetic rats, characterized by the normalized levels of tumour necrosis factor‐α (TNF‐α). In this connection, TNF‐α has been demonstrated to potentiate P2X_3_ receptor‐mediated nociception.^27^ A subsequent study by Xiong and colleagues further demonstrated that intrathecal injection of uc.48 + siRNA attenuated the upregulation of calcitonin gene‐related peptide, a peptide whose expression is associated with neuronal sensitization and enhanced pain,^37^ in the spinal cord of diabetic rats. These results suggested that targeting uc.48+ could alleviate diabetic neuropathic pain through suppressing the excitatory transmission regulated by P2X_3_ receptors in DRG.

### 3.3 NONRATT021972

NONRATT021972 is another lncRNA recently demonstrated to modulate P2X purinoceptors in diabetic neuropathic pain. It has been reported that the levels of NONRATT021972 were upregulated in the DRG of streptozotocin‐induced diabetic rats^38^ and Zucker diabetic fatty rats^39^ as well as in blood of patients with type 2 diabetes.^28^ Interestingly, circulating levels of NONRATT021972 were positively correlated with neuropathic pain scores in the latter study. Functionally, siRNA targeting NONRATT021972 restored sensory nerve conduction velocity and attenuated mechanical allodynia and thermal hyperalgesia in diabetic rats,^28^, ^38^, ^39^ indicating this lncRNA is pronociceptive. Similar to the studies on uc.48+, TNF‐α, P2X_3_ and ERK phosphorylation were all upregulated in DRG of diabetic rats, in which NONRATT021972 siRNA abolished such effects.^38^ Aside from neuronal P2X purinoceptors, NONRATT021972 siRNA was shown to attenuate P2X_7_ signalling in GFAP‐positive satellite glial cells, which presumably reduced TNF‐α levels.^39^ These findings collectively suggested that NONRATT021972 could mediate neuropathic pain through promoting both neuronal purinoceptor signalling and non‐neuronal purinoceptor signalling as well as enhancing the pro‐inflammatory state in diabetic neuropathic pain.

### 3.4 MRAK009713

Chronic constriction injury (CCI) is a common experimental model of neuropathic pain, in which the sciatic nerve is ligated resulting in inflammation and ultimately peripheral pain.^40^ Li and colleagues reported that the expression of a novel lncRNA MRAK009713 was upregulated in the DRG of CCI rats, in which siRNA silencing of this lncRNA alleviated mechanical allodynia and thermal hyperalgesia. Strikingly, overexpression of MRAK009713 was sufficient to induce neuropathic pain‐like symptoms.^41^ Computational prediction based on the secondary structure, hydrogen bonding and molecular interatomic forces suggested that MRAK009713 could bind to the P2X_3_ purinoceptor, in which the physical interaction was further confirmed by RNA immunoprecipitation assay. Enforced expression of MRAK009713 also upregulated the expression of P2X_3_ receptors and enhanced the inward current induced by α, β‐methylene‐adenosine‐5′‐triphosphate (a P2X_3_ agonist) in rat DRG.^41^ Collectively, these results indicated that MRAK009713 is a positive regulator of the neuropathic pain by enhancing P2X_3_ receptor expression and function.

### 3.5 XIST

X inactive specific transcript (XIST) is one of the best‐characterized lncRNAs to date owing to its heavy involvement in X inactivation. Several studies have demonstrated that XIST expression was upregulated in the spinal cord of CCI rats, in which knockdown of this lncRNA suppressed neuroinflammation and attenuated mechanical allodynia and thermal hyperalgesia.^42^, ^43^, ^44^ The pronociceptive action of XIST was found to be mediated through sponging of anti‐inflammatory microRNAs, namely miR‐137, miR‐150 and miR‐544, leading to derepression of their pro‐inflammatory targets.^42^, ^43^, ^44^ In particular, miR‐137 was found to target tumour necrosis factor alpha‐induced protein 1 (TNFAIP1), which is a crucial inflammation regulator by activating nuclear factor‐κB activity.^42^ Zinc finger E‐box‐binding homeobox 1 (ZEB1) and signal transducer and activator of transcription 3 (STAT3) were also identified as the targets of miR‐150 and miR‐544, respectively.^43^ In this regards, knockdown of ZEB1 has been shown to downregulate the expression of pro‐inflammatory cytokines, such as interleukin (IL)‐6 and IL‐8, in breast cancer,^45^ whereas STAT3 activation is known to stimulate spinal astrocyte proliferation to perpetuate neuropathic pain in rats. These results indicated that XIST promotes neuroinflammation to maintain neuropathic pain through the miR‐137/TNFAIP1, miR‐150/ZEB1 and miR‐544/STAT3 axes.

### 3.6 CCAT1

Colon cancer‐associated transcript‐1 (CCAT1) is a lncRNA transcribed from a distal enhancer 515‐kb upstream of the c‐*MYC* gene.^46^ Dou and colleagues^47^ demonstrated that CCAT1 was downregulated in anatomical structures along the nociceptive pathway, namely DRG, spinal dorsal horn, hippocampus and anterior cingulated cortex, in a rat model of bilateral CCI. Functional analysis of CCAT1 by overexpression indicated that CCAT1 could protect against mechanical allodynia in the neuropathic pain model. Further mechanistic study suggested that CCAT1 might mediate its antinociceptive effect through sponging miR‐155 and thereby derepressing serum and glucocorticoid regulated protein kinase 3 (SGK3). Concordantly, miR‐155 was upregulated, whereas SGK3 was downregulated in DRG, spinal dorsal horn, hippocampus and anterior cingulated cortex of rats sustained bilateral CCI.^47^ These results suggested that restored expression of CCAT1 or inhibition of miR‐155 might provide a novel therapeutic approach for the management of neuropathic pain (Table 2).

**Table 2:** Functional characterization of the lncRNAs in neuropathic pain

lncRNAs	Expression	Functional role	Related gene	References
KCNA2‐AS	Up	Reduced total Kv current and increased the excitability	Kcna2	26
uc.48+	Up	Enhanced mechanical allodynia and thermal hyperalgesia	P2X3 ERK	27
NONRATT021972	Up	Enhanced mechanical allodynia and thermal hyperalgesia	P2X3 ERK	28 37 38
MRAK009713	Up	Enhanced mechanical allodynia and thermal hyperalgesia	P2X3	40
XIST	Up	neuroinflammation and mechanical allodynia and thermal hyperalgesia	miR‐137, miR‐150, miR‐544	41, 42, 43
CCAT1	Down	mechanical allodynia	miR‐155	45
BC168687	up	mechanical allodynia and heat hyperalgesia	P2X7 TRPV1	47 48
NEAT1	Up	neuroinflammation	miR‐381	51

lncRNAs, long non‐coding RNAs.

### 3.7 BC168687

Liu and colleagues^48^ reported that the levels of the lncRNA BC168687 in the DRG of streptozotocin‐induced diabetic rats were significantly higher than that of the control group. In this connection, knockdown of BC168687 attenuated diabetes‐induced mechanical allodynia and heat hyperalgesia.^48^, ^49^ The increased expression levels of P2X_7_ on GFAP‐positive satellite glial cells in DRG and the elevated serum concentrations of nitric oxide (an oxidative injury factor released from satellite glial cells) in diabetic rats were also abrogated by BC168687 siRNA.^48^ A recent study by the same group of investigators further suggested that BC168687 could mediate its pronociceptive effect through enhancing the function and expression of transient receptor potential vanilloid type 1 (TRPV1; a nonselective cation channel that can be activated by capsaicin and ATP) expressed by DRG neurons in diabetic neuropathic pain. Concordantly, the elevated phosphorylation of ERK and p38 as well as the increased levels of TNF‐α and IL‐1β in the DRG of diabetic animals was abolished by BC168687 siRNA.^49^ These results suggested that BC168687 contributes to the pathogenesis of diabetic neuropathic pain by enhancing P2X_7_ and TRPV1 signalling in DRG satellite glial cells and neurons, respectively. Targeting BC168687 by siRNA or small molecules might be a promising approach to relieve neuropathic pain.

### 3.8 NEAT1

Nuclear paraspeckle assembly transcript 1 (NEAT1), transcribed from the familial tumour syndrome multiple endocrine neoplasia type 1 locus on chromosome 11, is a lncRNA localized to nuclear paraspeckles, which are subnuclear bodies found in the interchromatin space that regulate gene expression through RNA retention. Knockdown of NEAT1 has been shown to result in the disintegration of nuclear paraspeckles.^50^ Xia and colleagues^51^ reported that NEAT1 was significantly upregulated in the spinal cord of rats sustained CCI, in which knockdown of this lncRNA suppressed neuroinflammation (ie, reduced levels of IL‐1β, IL‐6 and TNF‐α) as well as attenuated mechanical allodynia and thermal hyperalgesia. miR‐381, whose levels were reduced in the spinal cord of CCI rats, was predicted to be a target of NEAT1. The physical interaction between NEAT1 and miR‐381 was further confirmed by luciferase reporter assay and RNA pull‐down assay. High‐mobility group box 1 (HMGB1), which is known to promote neuroinflammation,^52^ was identified as a direct target of miR‐381. Additional gain‐ and loss‐of‐function analyses indicated that NEAT1 modulated CCI‐induced neuropathic pain by regulating the miR‐381/HMGB1 axis.^51^ These results suggested that NEAT1 and HMGB1 are potential therapeutic targets in neural compression‐induced neuropathic pain (Table 2).

## 4 CONCLUSION

**Figure 1:**
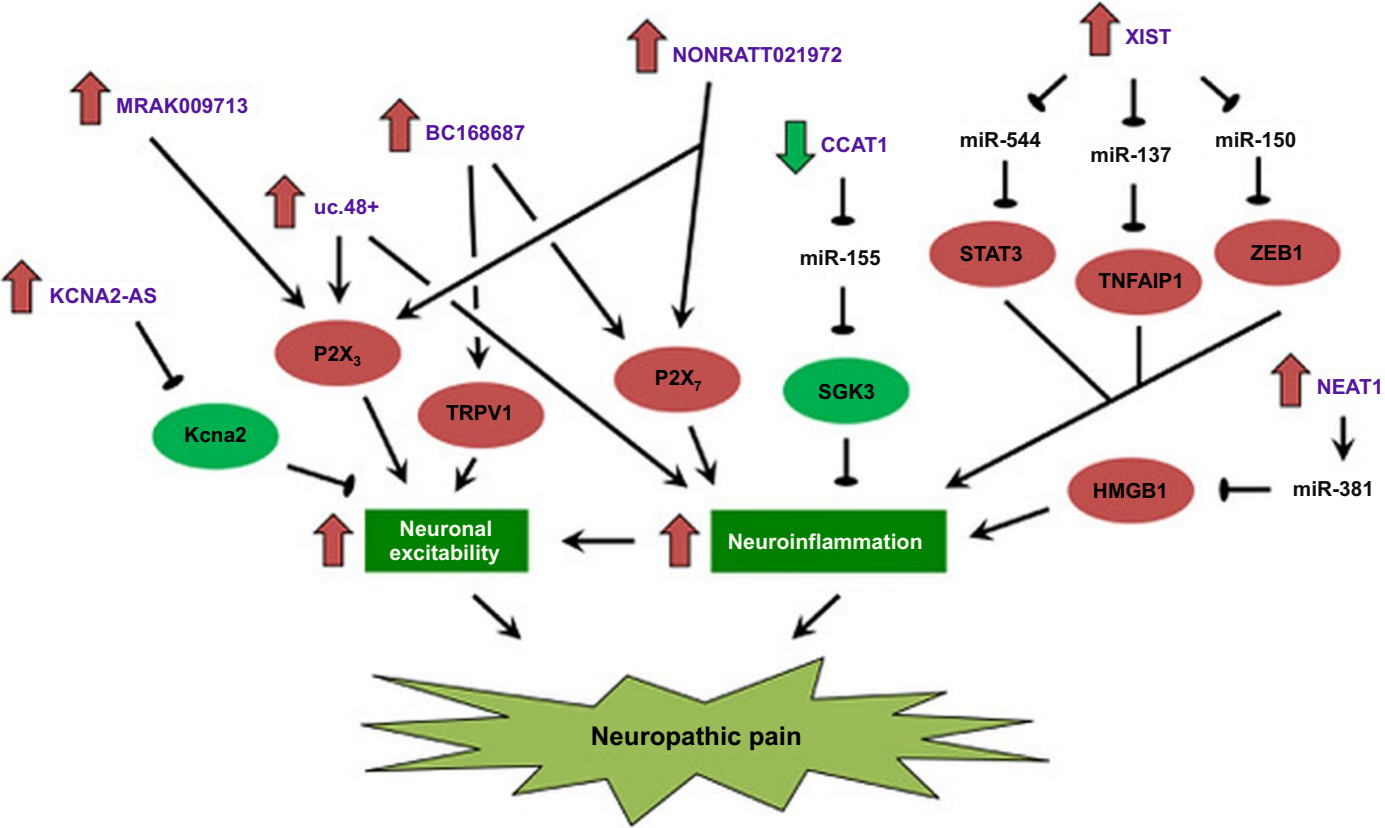
Deregulation of neuronal excitability and glia‐mediated neuroinflammation by lncRNAs in neuropathic pain

Neuropathic pain is a serious public health issue and an intractable clinical challenge, whose molecular mechanism is still largely elusive. Emerging studies suggested that lncRNAs play crucial roles in the development of neuropathic pain through regulating ion channels and neuroinflammation, two key features that drive the pathogenesis of neuropathic pain (Figure 1). From the mechanistic point of view, lncRNAs may regulate the gene expression of pain‐related molecules by binding to the mRNA (KCNA2‐AS) or sponging microRNAs (CCAT1, NEAT1 and XIST). Alternatively, lncRNA might directly bind to the target ion channel and potentiate the channel‐related current (MRAK009713). These studies have also shed new light on their potential clinical utility as therapeutic targets. In this regard, nucleic acid‐based therapeutics invoking multiple approaches, including (a) transcriptional inhibition through classical CRISPR/Cas9; (b) post‐transcriptional knockdown of lncRNAs by antisense oligonucleotides or small interfering RNAs; and (c) steric blockade of lncRNA‐protein interactions by small molecules and morpholinos, have been promulgated as a promising strategy to target pathogenic lncRNAs.^53^ Through RNA sequencing and RT‐qPCR, a growing number of deregulated lncRNAs are being identified and validated. Notably, differentially abundant circulating lncRNA could also be identified in the plasma of patients and positively correlated with pain scores, indicating that lncRNAs could be a new source of biomarkers for identifying and monitoring patients with neuropathic pain. However, further functional and mechanistic studies on these lncRNAs are warranted. Importantly, more translational studies are needed to maximize their use as biomarkers and therapeutic targets in neuropathic pain.

## ACKNOWLEDGEMENTS

This work was supported by Capital's funds for health improvement and research (2016–1–4096).
